# Ratio of Dietary n-6/n-3 Polyunsaturated Fatty Acids Independently Related to Muscle Mass Decline in Hemodialysis Patients

**DOI:** 10.1371/journal.pone.0140402

**Published:** 2015-10-14

**Authors:** Te-Chih Wong, Yu-Tong Chen, Pei-Yu Wu, Tzen-Wen Chen, Hsi-Hsien Chen, Tso-Hsiao Chen, Shwu-Huey Yang

**Affiliations:** 1 School of Nutrition and Health Sciences, Taipei Medical University, Taipei, Taiwan, R.O.C; 2 Division of Nephrology, Department of Internal Medicine, Taipei Medical University Hospital, Taipei, Taiwan, R.O.C; 3 Division of Nephrology, Department of Internal Medicine, Wan Fang Hospital, Taipei Medical University, Taipei, Taiwan, R.O.C; 4 Nutrition Research Center, Taipei Medical University Hospital, Taipei, Taiwan, R.O.C; University of Leicester, UNITED KINGDOM

## Abstract

**Background:**

n-3 polyunsaturated fatty acids (PUFAs) might be useful nutritional strategy for treating patients with sarcopenia. We evaluated the effect of the intake of dietary n-3 PUFAs on the skeletal muscle mass (SMM), appendicular skeletal muscle mass (ASM), and its determinants in patients receiving standard hemodialysis (HD) treatment for the management of end stage renal disease.

**Methods:**

In this cross-sectional study, data of 111 HD patients were analyzed. Anthropometric and bioelectrical impedance measurements used to estimate the muscle mass were performed the day of dialysis immediately after the dialysis session. Routine laboratory and 3-day dietary data were also collected. The cutoff value of adequate intake (AI) for both n-3 PUFAs and alpha-linolenic acid (ALA) was 1.6 g/day and 1.1 g/day for men and women, respectively.

**Results:**

The mean age, mean dietary n-3 PUFAs intake, ALA intake, ratio of n-6/n-3 PUFAs intake, SMM, and ASM of patients were 61.4 ± 10.4 years, 2.0 ± 1.3 g/day, 1.5 ± 1.0 g/day, 9.5 ± 6.7 g/day, 23.9 ± 5.5 kg, and 17.5 ± 4.5 kg, respectively. A higher SMM and ASM significantly observed in patients who achieved an AI of n-3 PUFAs. Similar trends appeared to be observed among those patients who achieved the AI of ALA, but the difference was not significantly, except for ASM (*P* = 0.047). No relevant differences in demographics, laboratory and nutritional parameters were observed, regardless of whether the patients achieved an AI of n-3 PUFAs. Multivariate analysis showed that the BMI and equilibrated Kt/V were independent determinants of the muscle mass. Moreover, the ratio of n-6/n-3 PUFAs was an independent risk determinant of reduced ASM in HD patients.

**Conclusion:**

Patients with an AI of n-3 PUFAs had better total-body SMM and ASM. A higher dietary ratio of n-6/n-3 PUFAs seemed to be associated with a reduced muscle mass in HD patients.

## Introduction

Sarcopenia, a generalized and progressive loss of skeletal muscle mass (SMM) and strength with age [1], is prevalent in chronic kidney disease (CKD) [2]. However, this pattern of change in body composition is related to factors associated with the potential causes of protein–energy wasting (PEW), the progressive and cumulative loss of muscle mass that occurs in CKD, such as inadequate food intake caused by poor appetite and dietary restrictions, systemic inflammation, comorbidities and complications, oxidative stress, and a decline in physical activity [3–5]. Findings from observational studies revealed that sarcopenia is highly prevalent in elderly hemodialysis (HD) patients [6]. Morishita et al. showed that sarcopenia increased the risk of fracture and reduced the quality of life in CKD patients [7]. Huang et al. asserted that a low SMM is related to increased mortality in patients receiving HD [8]. Overall, therapies designed to maintain muscle mass or reduce the metabolic derangements and other consequences associated with sarcopenia in HD patients might improve the physical exertion or survival of patients.

n-3 and n-6 polyunsaturated fatty acids (PUFAs) influence cell membrane integrity and potentially benefit muscle hypertrophy and atrophy [9]. n-3 PUFAs are rich in fish, flax and canola oil whereas n-6 PUFAs are found corn, sunflower, and safflower oils [9]. Alexander et al. demonstrated that scalded animals administered n-3 PUFAs instead of other lipids in the diet had an enhanced SMM [10]. Smith et al. revealed that dietary n-3 PUFA supplementation increased the rate of muscle protein synthesis in older adults [11]. By contrast, Candow et al. demonstrated that n-6 PUFAs, precursors to potent lipid mediator signaling molecules converted via sequential elongation and desaturation processes, may exacerbate the loss of muscle mass, and that the ratio of n-6/n-3 PUFAs plays a crucial role in the catabolic/anabolic reactions in muscle cells [9]. Considering the aforementioned findings, studies investigating the relationship between the dietary intake of n-3 and n-6 PUFAs and the maintenance of muscle mass are warranted in CKD.

Regarding dialysis patients, the beneficial effects of n-3 PUFAs supplementation on triglyceride levels, dialysis access patency, and possibly uremic pruritus or oxidative stress were described [12]. Currently, there is no well-established recommendation for n-3 PUFAs supplementation in people receiving dialysis. The American Heart Association (AHA) recommends the consumption of fish, preferably oily fish, at least twice weekly for healthy people [13]. The National Kidney Foundation (NKF) acknowledged the beneficial effects of consuming foods rich n-3 PUFAs, at least twice weekly, in well-nourished, stable dialysis patients whether they had evidence of cardiovascular disease (CVD) or not [14]. Nevertheless, Friedman et al. revealed that 67% of HD patients did not meet the AHA fish-consumption guidelines for healthy people and had low plasma n-3 PUFA levels [15]. The authors also asserted that HD patients are an ideal group in which to demonstrate the effects of n-3 PUFAs supplementation [16]. To the best of our knowledge, published reports have discussed the beneficial effects of n-3 PUFAs in HD patients, focusing primarily on anti-inflammation and nutritional markers [17–19]. Cano et al. highlighted that no studies have reported the effects of n-3 PUFAs intake on muscle cell metabolism in HD patients [20].

By considering the health benefits provided by n-3 PUFAs in the general population, Sakuma and Yamaguchi asserted that an adequate intake (AI) of n-3 PUFAs is 1.6 g/day and 1.1 g/day for men and women, respectively [21]. However, the US Institute of Medicine (IOM) suggested that alpha-linolenic acid (ALA) was the only true “essential” n-3 PUFAs in the diet [22]. Thus, the aim of the present study was to investigate whether muscle mass is associated with dietary n-3 PUFAs and ALA in patients receiving HD. We also attempted to determine the possible univariate significant or nonsignificant relevant determinants of muscle mass in HD patients.

## Patients and Methods

### Study design and participants

**Fig 1:**
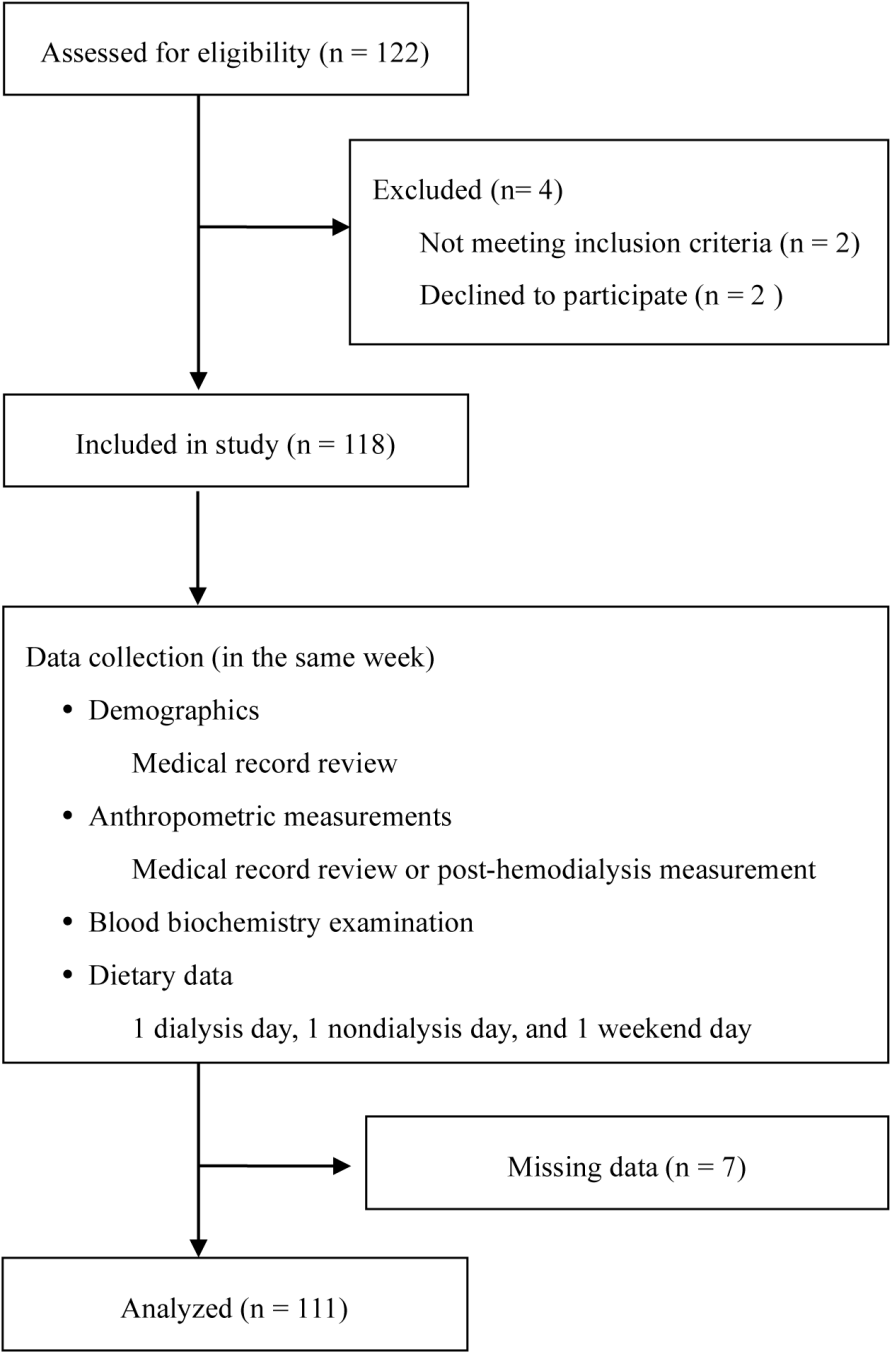
Flow chart indicates patient enrolment and study procedure.

This cross-sectional study involved the HD Centers at Taipei Medical University Hospital (TMUH) and Wan Fang Hospital (WFH). From September 2013 to May 2014, 122 patients undergoing HD for at least 3 months were enrolled. The recruitment criteria required participants be aged ≥20 years and receiving regular HD treatment with a target dialysis dose expressed as equilibrated Kt/V (eKt/V) of at least 1.2 in the previous 3 months. Exclusion criteria were as follows: (i) patients whose anthropometric, clinical biochemistry, and dietary data were missing; (ii) patients whose energy intake was <500 kcal/day or >3500 kcal/day; or (iii) patients with obvious edema, amputation, hyperthyroidism, hypothyroidism, malign tumor, hospitalization or who were pregnant (Fig 1). The study was approved by the Research Ethics Committee of Taipei Medical University (201302024), and written informed consent was obtained from all study participants (S1 File).

### Data collection

Data were collected from the patients by the well-trained staff according to standardized methods and procedures. Patient demographics and anthropometric and blood biochemical values were recorded at baseline in the same week.

#### Demographics and anthropometric measurements

By reviewing patient medical charts, we collected demographic data such as age; sex; dialysis vintage and dose; and history of diabetes, hypertension, and CVD. The presence of comorbidities was evaluated using the Charlson comorbidity index [23]. Anthropometric information, including height, dry weight, and interdialytic weight gain, was also collected from the chart review. Body mass index (BMI) was calculated as post-dialysis weight (kg)/[height (m)]^2^. Muscle mass such as SMM, and appendicular skeletal muscle mass (ASM) were estimated through bioelectrical impedance analysis (BIA) (InBody S10, Biospace, Seoul, Korea). This analyzer measures segmental impedances at trunk, the right arm, left arm, right leg, and left leg using multiple operating frequencies of 1, 5, 50, 250, 500, and 1,000 kHz. Measurements were taken when the patients were a sitting position after HD. In this study, SMM and ASM were estimated as the sum of total body muscle mass and the mass of the four limbs respectively. Moreover, SMM and ASM were normalized for body height as the SMM index and the ASM index for the definition of body composition indices, respectively.

#### Blood biochemistry examination

In this study, 8-hour preprandial and predialysis blood samples were withdrawn and subsequently analyzed in the clinical laboratories of TMUH or WFH. All biochemistry examinations were performed using automated and standardized methods. The following parameters were analyzed: albumin (bromocresol green), total cholesterol, hemoglobin, creatinine, insulin, high-sensitivity C-reactive protein (hs CRP), and homocysteine. In this study, homoeostasis model assessment-estimated insulin resistance (HOMA-IR) was used as an index of insulin resistance and calculated as (Glucose) × (Insulin) / 405 (glucose in mg/dL) [24]. In addition, geriatric nutritional risk index (GNRI), a simplified nutritional screening index, was calculated as described previously [25]. For both sexes, cutoff values for hypoalbuminemia, low-grade inflammation, and nutritional risk were albumin < 3.5 g/dL, hs CRP > 0.5 mg/dL [26], and GNRI < 91.2 [25], respectively.

#### Dietary data

Detailed procedures for collecting dietary data have been published previously [27]. Briefly, all patients completed 3-day dietary records (1 dialysis day, 1 nondialysis day, and 1 weekend day) before they visited the well-trained dietitian. A 24-hour dietary recall was conducted via face-to-face or phone interviews with the patients to confirm the dietary data. Energy and nutrient intake were estimated by using Nutritionist Edition, Enhancement Plus 3, Version 2009, a nutrient analysis software containing a Taiwanese food composition table as the nutrient database (Taichung, Taiwan). The consumption of total calories and proteins, normalized according to body weight, saturated fatty acids (SFAs), monounsaturated fatty acids (MUFAs), and PUFAs, as well as the sum of n-3 PUFAs and n-6 PUFAs were investigated. The ratios of n-3/n-6 PUFAs and n-6/n-3 PUFAs were also calculated. In this study, the AI of n-3 PUFAs was 1.6 g/day and 1.1 g/day for men and women, respectively, according to previous studies [21, 22].

#### Physical activity

The short version of the International Physical Activity Questionnaire (IPAQ) was used in this study; its details were described previously [28]. This self-description questionnaire was completed by the interviewer. The average number of days and the average duration a patient spent exercising (vigorous, moderate, or walking exercise) over the previous 7 days were recorded. The amount of occupational sitting time during the weekdays was also determined. A metabolic equivalent (MET) value was assigned according to sleep and four levels of physical activity (light, moderate, intense, and very intense) and reported in kcal/day.

### Statistical analyses

Statistical analyses were conducted using SAS software (SAS Version 9.3). Data were presented as the mean ± standard deviation, percentage, regression coefficient with 95% confidence intervals (CIs) or r square (R^2^) as appropriate. The Shapiro–Wilk test was used to assess normality. The patients were divided into two groups according to whether or not the AI of n-3 PUFAs was achieved, and the results were compared using the Student’s t test, Wilcoxon rank sum test, or Chi-square test as appropriate. Simple linear regression was applied to identify the predictors of SMM or ASM. Considering the ratio of n-6/n-3 PUFAs, the distributions were skewed and thus we deleted the extreme values (outliers) using the box-and-whisker plots to identify its correlations with muscle mass. Furthermore, multiple linear regression analysis was performed to investigate the effect of dietary n-3 PUFAs or n-6 PUFAs on the muscle mass. Considering the total dietary intake of n-3 PUFAs and ALA, we investigated only n-3 PUFAs as an independent variable in our multivariate model because of the expected high collinearity that ALA can be linearly predicted with a substantial degree of accuracy. In the multivariable models, we selected adjustment factors by considering that they significantly affected the muscle mass, as shown in the univariate study, and were known prognostic factors. The different variable-adjusted models were (i) model A: age and sex; (ii) model B: age, sex, and total energy and fat intake; and (iii) multivariate B: significant variables (*P* < 0.05) according to model B.

## Results

### Patient characteristics

Table 1 showed the demographic, anthropometric, laboratory, and clinical characteristics of the 111 HD patients (56 men and 55 women). The mean age and the dialysis vintage of patients were 61.4 ± 10.4 years (range: 27–86 years) and 5.4 ± 5.3 years (range: 3.6 months–32.3 years), respectively. Patients received an adequate dialysis dose according to the eKt/V. The presence of comorbidities and the nutritional status were expressed as scores of 2.9 ± 0.8 and 101 ± 4.8 according to the Charlson comorbidity index and GNRI, respectively.

**Table 1:** Demographic, anthropometric, clinical and nutritional characteristics of the 111 HD subjects^1^ ^,^ ^2^.

	All			Omega-3 intake^3^						ALA intake^4^					
				≧ AI			< AI			≧ AI			< AI		
n	111			71			40			57			54		
**Demographics**															
Male/female	56/55			37/34			19/21			30/27			26/28		
Age, years	61.4	±	10.4	60.6	±	9.5	62.9	±	11.7	60.4	±	10.2	62.5	±	10.5
Dialysis vintage, year	5.4	±	5.3	5.2	±	4.7	5.8	±	6.2	5.7	±	5.1	5.1	±	5.5
Diabetes, n (%)	57 (51.4)			36 (50.7)			21 (52.5)			41 (71.9)			37 (68.5)		
Hypertension, n (%)	39 (35.1)			26 (36.6)			13 (32.5)			20 (35.1)			19 (35.2)		
History of CVD, n (%)	64 (57.7)			43 (60.6)			21 (52.5)			38 (66.7)			26 (48.1)		
Charlson comorbidity index	2.9	±	0.8	2.9	±	0.8	2.9	±	0.9	3.0	±	0.8	2.8	±	0.8
**Anthropometry**															
Height, cm	161.8	±	8.5	162.9	±	8.4	159.7	±	8.3	163.2	±	8.4	160.2	±	8.5
Body weight, kg	61.4	±	12.2	62.8	±	12.9	59.0	±	10.4	63.0	±	13.7	59.7	±	10.2
BMI, kg/m^2^	23.4	±	3.6	23.5	±	3.7	23.1	±	3.5	23.5	±	4.0	23.2	±	3.2
SMM, kg	23.9	±	5.5	24.7	±	6.0	22.6	±	4.4†	24.8	±	6.2	23.0	±	4.5
SMM index, kg/m^2^	9.1	±	1.4	9.2	±	1.5	8.8	±	1.2	9.2	±	1.6	8.9	±	1.2
ASM, kg	17.5	±	4.5	18.2	±	4.8	16.2	±	3.7†	18.3	±	4.9	16.6	±	4.0‡
ASM index, kg/m^2^	6.6	±	1.2	6.7	±	1.2	6.3	±	1.0†	6.8	±	1.3	6.4	±	1.0
**Laboratory**															
Alb, g/dL	4.1	±	0.3	4.1	±	0.3	4.1	±	0.3	4.1	±	0.3	4.1	±	0.3
TC, mg/dL	169.9	±	36	168	±	36.2	173.3	±	35.7	169.5	±	36.5	170.3	±	35.7
Hb, g/dL	10.7	±	1.2	10.7	±	1.2	10.8	±	1.1	10.6	±	1.2	10.8	±	1.1
Creatinine, mg/dL	11.2	±	2.0	11.4	±	1.8	11.0	±	2.3	11.3	±	2.0	11.1	±	2.1
Insulin, μU/mL	17.1	±	13.7	16.0	±	13.5	19.1	±	14.1	15.1	±	12.4	19.3	±	14.7
hs CRP, mg/dL	0.5	±	0.8	0.5	±	0.8	0.6	±	0.7	0.4	±	0.6	0.6	±	1.0
Homocystein, μmol/L	20.2	±	7.7	19.8	±	7.9	20.8	±	7.4	20.7	±	7.7	19.7	±	7.8
**Dietary intake**															
Energy, kcal/day	1675.7	±	455.9	1728.7	±	442.7	1581.7	±	469.3†	1768.8	±	434.9	1577.5	±	460.9‡
Energy, kcal/kg/day	28.2	±	9.5	28.6	±	9.6	27.6	±	9.3	29.4	±	10.1	27.0	±	8.7
Protein, g/day	61.1	±	22.3	64.0	±	20.9	56.0	±	24.1	65.1	±	21.3	56.9	±	22.7
Protein, g/kg/day	1.0	±	0.4	1.1	±	0.4	1.0	±	0.5	1.1	±	0.4	1.0	±	0.4
Total fat, g/day	66.1	±	26	73.1	±	22.3	53.6	±	27.6†	76.0	±	20.8	55.6	±	27‡
Total PUFA, g/day	17.7	±	9.5	22.0	±	8.5	10.1	±	5.6†	24.1	±	7.8	10.9	±	5.5‡
Total MUFA, g/day	21.1	±	10.7	23.6	±	10.2	16.8	±	10.4†	23.3	±	9.7	18.9	±	11.4‡
Total SFA, g/day	15.9	±	7.6	18.4	±	6.4	11.5	±	7.6†	18.6	±	6.1	13.1	±	8.0‡
ALA, g/day	1.5	±	1.0	2.0	±	0.8	0.6	±	0.4†	2.2	±	0.8	0.7	±	0.4‡
Total n-3 PUFA, g/day	2.0	±	1.3	2.7	±	1.2	0.8	±	0.4†	2.9	±	1.0	1.1	±	0.9‡
Total n-6 PUFA, g/day	15.7	±	8.5	19.3	±	7.7	9.3	±	5.4†	21.3	±	7.0	9.8	±	5.2‡
Ratio of n-3/n-6 PUFA, g/day	0.1	±	0.1	0.1	±	0.1	0.1	±	0.1†	0.1	±	0.0	0.1	±	0.2
Ratio of n-6/n-3 PUFA, g/day	9.5	±	6.7	7.6	±	2.2	13.0	±	10.1†	7.8	±	1.8	11.4	±	9.2‡
**Others**															
HOMA-IR	5.2	±	5.2	5.0	±	5.4	5.7	±	4.9	4.6	±	4.4	6.0	±	5.9
GNRI	101	±	4.8	101.2	±	5.0	100.7	±	4.6	101	±	5.1	101.1	±	4.5
eKt/V	1.6	±	0.3	1.6	±	0.3	1.6	±	0.3	1.6	±	0.3	1.6	±	0.3
MET per day	566.5	±	264.4	582.2	±	254.2	538.5	±	282.6	589.1	±	249.3	542.6	±	279.7

Abbreviations: CVD, cardiovascular disease; BMI, body mass index; SMM, skeletal muscle mass; ASM, appendicular skeletal muscle mass; Alb, albumin; TC, total cholesterol; Hb, hemoglobin; hs CRP, high-sensitivity C reactive protein; PUFA, polyunsaturated fatty acid; MUFA, monounsaturated fatty acid; SFA, saturated fatty acid; ALA, alpha-linolenic acid; P:M:S, polyunsaturated: monounsaturated: saturated; HOMA-IR, homoeostasis model assessment-estimated insulin resistance, GNRI, geriatric nutritional risk index; MET, metabolic equivalent; eKt/V: equilibrated Kt/V; AI, adequate intake

^1^Values are shown as the mean ± standard deviation or percentage, as appropriate.

^2^Statistical analyses were conducted using Student’s *t* test, Wilcoxon rank sum test, or Chi-square test

†*P* < 0.05 in comparison with AI of omega-3 group

‡*P* < 0.05 in comparison with AI of ALA group

^3,4^ The cutoff value of adequate intake for n-3 fatty acids was 1.6 g/d and 1.1 g/d for men and women, respectively, according to previous studies [21, 22].

### Comparison of patients with AI of n-3 PUFAs or not (Table 1)

Of the 111 HD patients, the mean consumption of dietary ALA, n-3 PUFAs, and n-6 PUFAs were 1.5 ± 1.0 g/day, 2.0 ± 1.3 g/day, and 15.7 ± 8.5 g/day, respectively. Considering the AI of n-3 PUFAs, 33.3% male (n = 37) and 30.6% female (n = 34) were obtained and the mean intake of the n-3 PUFAs were 3.2 ± 1.1 g/day and 2.1 ±0.9 g/day respectively. In addition, these patients had a significantly higher total energy and fat intake, ratio of n-3/n-6 PUFAs, SMM, ASM, and ASM index than those who did not achieve the AI of n-3 PUFAs. Patients achieving the AI of ALA had similar trends to those of patients achieving the AI of n-3 PUFAs, but the difference was not significant, except for ASM (*P* = 0.047). Moreover, no relevant differences in total protein intake; demographical and laboratory findings; BMI, and physical activity level were observed, regardless of whether the AI of n-3 PUFAs or ALA was achieved.

### Identified confounders

**Fig 2:**
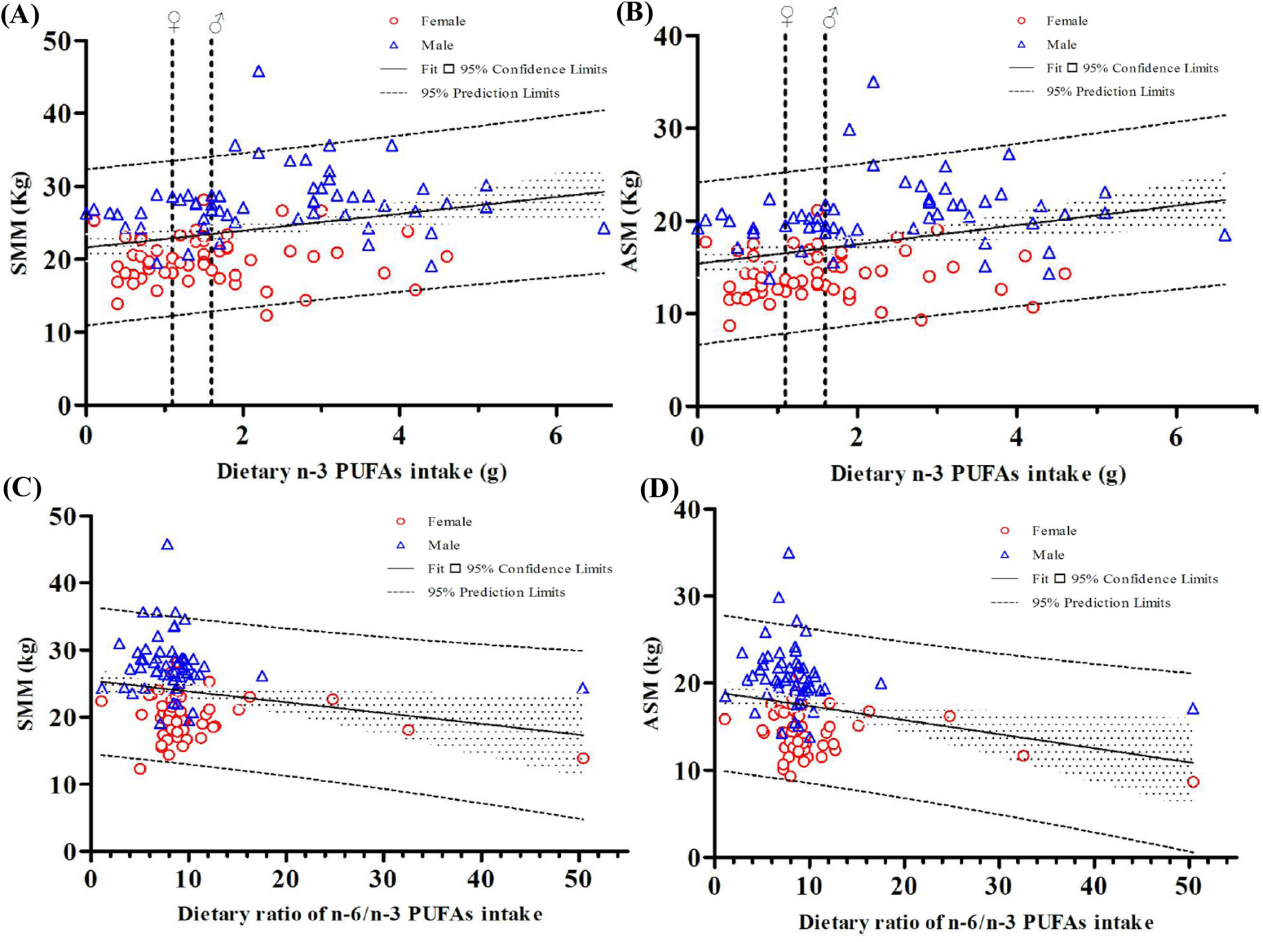
Changes in the muscle mass were related the dietary n-3 fatty acids and the ratio of n-6/n-3 PUFAs. The cutoff value of adequate intake for n-3 fatty acids was 1.6 g/d for men and 1.1 g/d for women (bold dotted line). The regression coefficients with 95% confidence intervals were 1.160 (0.396 to 1.924 kg) for (A), 1.043 (0.419 to 1.667 kg) for (B), −0.161 (−0.315 to −0.007 kg) for (C), and −0.162 (−0.287 to −0.036 kg) for (D). Abbreviations: SMM, skeletal muscle mass; ASM, appendicular skeletal muscle mass; PUFA, polyunsaturated fatty acid

Factors affecting the muscle mass were examined by performing simple regression analysis (Table 2). Both SMM and ASM were positively correlated with BMI, creatinine, and dietary n-3 PUFA intake (Fig 1) and were negatively correlated with eKt/V and the ratio of n-6/n-3 PUFAs (Fig 2). No significant correlation was found between muscle mass and dialysis vintage, insulin resistance, or CRP.

**Table 2:** Simple linear regression analysis using muscle mass (SMM or ASM) as dependent variable in 111 HD subjects^1^.

	SMM				ASM			
	β	95% CI	R^2^	*P* value	β	95% CI	R^2^	*P* value
**Univariate significant**								
n-3 PUFA intake, g/day	1.160	0.396 to 1.924	0.077	0.003	1.043	0.419 to 1.667	0.091	0.001
Ratio of n-6/n-3 PUFA, g/day	-0.161	-0.315 to -0.007	0.038	0.041	-0.162	-0.287 to -0.036	0.057	0.012
BMI, kg/m^2^	0.721	0.467 to 0.976	0.225	< .0001	0.576	0.365 to 0.788	0.211	< .0001
Creatinine, mg/dL	1.257	0.797 to 1.717	0.212	< .0001	1.011	0.629 to 1.393	0.201	< .0001
eKt/V	-11.014	-13.561 to -8.468	0.402	< .0001	-9.052	-11.155 to -6.949	0.395	< .0001
**Nonsignificant relevant covariates**								
Dialysis vintage, year	-0.183	-0.377 to 0.012	0.031	0.066	-0.155	-0.315 to 0.006	0.032	0.059
HOMA-IR	0.124	-0.075 to 0.323	0.014	0.220	0.104	-0.060 to 0.268	0.014	0.213
hs CRP, mg/dL	1.092	-0.236 to 2.420	0.023	0.106	1.080	-0.009 to 2.169	0.034	0.052

Abbreviations: SMM, skeletal muscle mass; ASM, appendicular skeletal muscle mass; HD, hemodialysis; BMI, body mass index; PUFA, polyunsaturated fatty acid; eKt/V: equilibrated Kt/V; HOMA-IR, homoeostasis model assessment-estimated insulin resistance; hs CRP, high-sensitivity C reactive protein; CI, confidence interval

^1^β refers to the regression coefficient that demonstrated the change in muscle mass per kg change in the exposure variable

We found few data points of the n-6/n-3 PUFA ratio illustrated at outliers (Fig 2). After outliers removed, significant correlations were still found between the ratio of n-6/n-3 PUFAs and muscle mass negatively (β_SMM_ = −0.592, *P* = 0.05; β_ASM_ = −0.645, *P* = 0.01).

### Independent factors associated with muscle mass on HD patients

The multiple linear regressions were performed in steps and designed to select factors independently associated with the muscle mass (Table 3). We found that SMM exhibited significant and independent associations with the history of CVD (β = −1.355), BMI (β = 0.357), and eKt/V (β = −3.791) (adjusted R^2^ = 0.693, *P* < 0.0001). In addition, BMI (β = 0.275), creatinine (β = 0.342), eKt/V (β = −3.058), and the ratio of n-6/n-3 PUFAs (β = −0.024) were independent variables significantly associated with ASM (adjusted R^2^ = 0.712, *P* < 0.0001).

**Table 3:** Multiple linear regression analysis using muscle mass (SMM or ASM) as dependent variable in 111 HD subjects^1^.

	Model A^※^			Model B^†^			Multivariate B^‡^		
	β	95% CI	*P* value	β	95% CI	*P* value	β	95% CI	*P* value
SMM									
CVD	-1.465	-0.002 to -2.927	0.050	-1.488	-0.02 to -2.949	0.046	-1.355	-0.074 to -2.637	0.038
BMI, kg/m^2^	0.524	0.352 to 0.696	< .0001	0.510	0.335 to 0.685	< .0001	0.357	0.175 to 0.540	0.0002
Creatinine, mg/dL	0.514	0.137 to 0.892	0.008	0.526	0.151 to 0.901	0.006	0.328	-0.008 to 0.665	0.056
eKt/V	-5.258	-7.817 to -2.699	< .0001	-5.062	-7.736 to -2.388	0.0003	-3.791	-6.366 to -1.216	0.004
MET per day	0.003	0.0003 to 0.006	0.026	0.003	0.0003 to 0.006	0.025	0.001	-0.001 to 0.003	0.374
n-3 PUFA intake, g/day	0.012	-0.011 to 0.036	0.292	0.019	-0.009 to 0.047	0.179			
n-6 PUFA intake, g/day	0.009	-0.014 to 0.032	0.437	0.016	-0.011 to 0.044	0.243			
Ratio of n-3/n-6 PUFA	0.008	-0.023 to 0.040	0.607	0.006	-0.026 to 0.038	0.722			
Ratio of n-6/n-3 PUFA	-0.013	-0.036 to 0.009	0.241	-0.012	-0.035 to 0.010	0.286			
ASM									
BMI, kg/m^2^	0.417	0.275 to 0.560	< .0001	0.404	0.260 to 0.548	< .0001	0.275	0.128 to 0.421	0.0003
Creatinine, mg/dL	0.420	0.111 to 0.730	0.008	0.431	0.124 to 0.737	0.006	0.342	0.072 to 0.613	0.014
eKt/V	-4.228	-6.332 to -2.124	0.0001	-4.051	-6.244 to -1.858	0.0004	-3.058	-5.089 to -1.027	0.004
MET per day	0.002	0.00009 to 0.004	0.041	0.002	0.000 to 0.004	0.041	0.001	-0.001 to 0.003	0.318
n-3 PUFA intake, g/day	0.015	-0.004 to 0.034	0.125	0.021	-0.002 to 0.043	0.071			
n-6 PUFA intake, g/day	0.005	-0.014 to 0.024	0.591	0.009	-0.014 to 0.031	0.453			
Ratio of n-3/n-6 PUFA	0.020	-0.005 to 0.046	0.121	0.018	-0.008 to 0.044	0.170			
Ratio of n-6/n-3 PUFA	-0.021	-0.039 to -0.003	0.026	-0.020	-0.038 to -0.001	0.035	-0.024	-0.039 to -0.008	0.003

Abbreviations: SMM, skeletal muscle mass; ASM, appendicular skeletal muscle mass; HD, hemodialysis; CVD, cardiovascular disease; BMI, body mass index; PUFA, polyunsaturated fatty acid; eKt/V: equilibrated Kt/V; MET: metabolic equivalent; CI, confidence interval

^1^β refers to the regression coefficient that the change in muscle mass per kg change in the exposure variable

^**※**^Model A: adjusted age and sex

^**†**^Model B: adjusted age, sex, total energy and fat intake

^**‡**^Multivariate B: adjusted for the factors with *P* < 0.05 according to Model B

## Discussion

We demonstrated that dietary n-3 PUFAs, highly related to ALA intake, corresponded to the muscle mass in patients receiving HD (Table 1 and Fig 2). This result indicates that dietary fatty acid intake may be a regulator of muscle mass. In the multiple linear regression analysis, BMI and eKt/V were the independent determinants of muscle mass. Furthermore, history of CVD and the ratio of n-6/n-3 PUFAs were independent risk factors for SMM and ASM, respectively (Table 3). In this study, the estimated dietary PUFA intakes do not give a full picture of overall nutritional adequacy. The results we observed are more likely to be a direct and specific effect of such fatty acids on muscle protein metabolism. Further studies are warranted to verify our preliminary results.

Interest on the beneficial effects of n-3 PUFAs on body composition change is growing. Muscle mass may decline 1–2% per year after age 50 years [29].The results of this study are consistent with those of Cornish et al. [30]: n-3 PUFAs, indicated as ALA, were associated with a greater increase in the thickness of the muscle mass in older adult males. These positive correlations between muscle mass and dietary n-3 PUFAs implies a therapeutic n-3 FA dietary intervention (a few g per day) which would presumably increase muscle mass by (at most) a few Kg. The possible mechanisms underlying the biological effects of PUFAs on the muscle mass are multifactorial. The anti-inflammatory properties of n-3 PUFAs may reduce the production of inflammatory cytokines to attenuate the anabolic resistance of muscle protein synthesis [31]. In addition, a high ratio of n-6/n-3 PUFAs in the diet promotes inflammatory diseases [32] that may interfere in the translation for the synthesis of SMM [33] or may break down the muscle protein [34].

We were unable to reveal a relationship between CRP levels and muscle mass in the multiple regression analysis. Similar to the current results, Schaap et al. found no associations between CRP and relative changes in the muscle mass of 328 older adults in a follow-up study [35]. The predominant characteristics of our patients were a healthier nutritional status and less comorbid conditions; 1.8% of the patients (n = 2) had albumin of <3.5 g/dL, 4.5% of the patients (n = 5) had a GNRI of <91.2, 32.4% of the patients (n = 36) had CRP levels of at least 0.5 mg/dL, and 72.1% (n = 80) of the patients had a Charlson comorbidity index score of ≤3 [36]. It would be interesting to investigate whether other inflammatory markers is associated with PUFA-related maintenance of muscle mass.

We found no relationship between dialysis vintage and muscle mass among the patients, even though a previous study revealed that the loss of SMM was negatively associated with dialysis vintage [37]. The inconsistency in the results of the relationship between dialysis vintage and muscle mass can be explained as follows: the HD patients in our study (i) were relatively young (61.4 ± 10.4 years versus 66.9 ± 9.6 years), (ii) had a relatively short dialysis vintage (5.4 ± 5.3 years versus 6.3 ± 4.7 years), and (iii) had a high SMM index, analyzed using a similar BIA equipment (9.1 ± 1.4 kg/m^2^ versus 4.6 ± 1.0 kg/m^2^). Although we considered several prevalent factors regarding muscle mass in the HD patients, the cross-sectional design of our study might have confounded the observed phenomenon.

The present study results also indicated that BMI, creatinine, and a history of CVD were independently related to the muscle mass. Lower muscle mass indicated as the low excretion of creatinine related to adverse cardiovascular events in the general population [38]. Kaynar et al. found a significant positive correlation between BMI and muscle mass [39]. These studies suggest that creatinine and BMI were the primary nutritional indicators determining the association between lower serum albumin and higher mortality risk among HD patients [40].

The Kt/V results in the present study were associated with a loss of ASM in the patients. These results were consistent with those of Morishita et al., who demonstrated that Kt/V was negatively associated with both muscle mass and handgrip strength in 34 HD patients [37]. It implies that patients with a low muscle mass may require a higher clearance in relation to the volume of urea distribution. We strongly recommend that Kt/V should be carefully estimated and SMM should be considered for adequate dialysis.

Several strengths and limitations of our study must be addressed when interpreting the results. According to the nutritional and anthropometric parameters, the patients were healthier and more physically capable than were the general HD population. Further studies are required to assess whether our observations can be extrapolated to other HD patients with different ethnicity, modality of renal replacement therapy, or status of chronic inflammation and nutrition wasting. In this study, we used noninvasive and easily available BIA to measure of muscle mass instead of dual-energy X-ray absorptiometry, computed tomography, or magnetic resonance imaging (MRI), the gold standard, but not readily performable at all medical institutions. Additionally, BIA results of estimation of the limb muscle mass in HD patients correlate closely with those obtained using MRI [41]. Determining the recommendations for cutoff points of muscle mass, or the rate of muscle loss in HD patients merits further research. Furthermore, we did not directly measure plasma and erythrocyte fatty acid levels. In our previous study [42], the plasma fatty acid composition of 16 diabetic patients analyzed through gas chromatography corresponded to the fish consumption patterns. Svensson et al. investigated the clinically meaningful findings that a self-reported dietary n-3 PUFAs associated with plasma lipid levels in HD patients [43]. Future studies should be performed to elucidate the possible relationship between muscle mass and fatty acid compositions in plasma and erythrocytes in patients receiving HD. Finally, in this cross-sectional study, we may have been unable to determine the causal relationships and statistical differences between n-3 PUFAs and muscle mass because of other unmeasured or residual confounding factors, such as the relatively small number of patients, the variation in dietary pattern and overall nutritional adequacy caused by geographical differences or socioeconomic status.

In conclusion, we revealed the relationship between dietary PUFAs and muscle mass and demonstrated that dietary n-3 PUFAs are determinants of the muscle mass in HD patients. Certain conditions associated with HD, such as dialysis dose and CVD, are negatively associated with muscle mass. In addition, the ratio of n-6/n-3 PUFAs is an independent risk factor for ASM. Based on our findings, increasing higher quantities of n-3 PUFAs in diet is one approach to normalizing higher ratio of n-6/n-3 PUFAs, characteristic of the Western diet and the possible risk factor for muscle mass in HD patients. Although the mechanisms by which n-3 PUFAs improve and maintain muscle homeostasis remain unclear, based on the evidence-based dietetic practice, our study introduces novel strategies to alleviate sarcopenia.

## Supporting Information

S1 File The certification of Taipei Medical University Joint Institutional Review Board. (DOCX)
