# Supplementary material for: Ratio of Dietary n-6/n-3 Polyunsaturated Fatty Acids Independently Related to Muscle Mass Decline in Hemodialysis Patients
Source: PLoS One. 2015 Oct 14;10(10):e0140402. doi: 10.1371/journal.pone.0140402 (PMC4605692; doi:10.1371/journal.pone.0140402)
Supplement: S1 File — (DOCX) [file pone.0140402.s001.docx]

S1 Appendix. The certification of Taipei Medical University Joint Institutional Review Board
